# Protective Effects of N^1^-Methylnicotinamide Against High-Fat Diet- and Age-Induced Hearing Loss *via* Moderate Overexpression of Sirtuin 1 Protein

**DOI:** 10.3389/fncel.2021.634868

**Published:** 2021-04-06

**Authors:** Toru Miwa

**Affiliations:** ^1^Department of Otolaryngology and Head and Neck Surgery, Kitano Hospital, Tazuke Kofukai Medical Research Institute, Osaka, Japan; ^2^Department of Otolaryngology and Head and Neck Surgery, Graduate of School of Medicine, Kyoto University, Kyoto, Japan

**Keywords:** hearing loss, sirtuin 1, N^1^-methylnicotinamide, high-fat diet, auditory brain stem responses

## Abstract

Age-related hearing loss (ARHL) is the most common form of hearing loss and the predominant neurodegenerative disease associated with aging. *Sirtuin 1 (SIRT1)* is associated with the most complex physiological processes, including metabolism, cancer onset, and aging. SIRT1 protein levels are enhanced by the conversion of nicotinamide to N^1^-methylnicotinamide (MNAM), independent of its mRNA levels. Moreover, MNAM has implications in increased longevity achieved through its mitohormetic effects. Nicotinamide N-methyltransferase (Nnmt) is an enzyme involved in MNAM metabolism, and its level increases under caloric restriction (CR) conditions. The CR condition has implications in delaying ARHL onset. In this study, we aimed to determine the relationship between diet, hearing function, SIRT1 and SIRT3 expression levels in the inner ear, and cochlear morphology. Mice fed with a high-fat diet (HFD), HFD + 1% MNAM, and low-fat diet (LFD) were monitored for age-related auditory-evoked brainstem responses, and changes in cochlear histology, metabolism, and protein and mRNA expressions were analyzed. Our results revealed that the HFD- and aging-mediated downregulated expression of SIRT1 and SIRT3 promoted hearing loss that was obfuscated by MNAM supplementation-induced upregulated expression of cochlear SIRT1 and SIRT3. Thus, our results suggest that MNAM can be used as a therapeutic agent for preventing ARHL.

## Introduction

Age-related hearing loss (ARHL) is a multifactorial disease that results from a combination of genetic predispositions and effects of the aging process, including a plethora of insults to the auditory organ throughout an individual’s lifetime (Gates and Mills, 2005; Yamasoba et al., 2013). ARHL can be prevented by improving the health and quality of life in elderly individuals; however, thus far, no preventive measures have been outlined.

**Figure 1:**
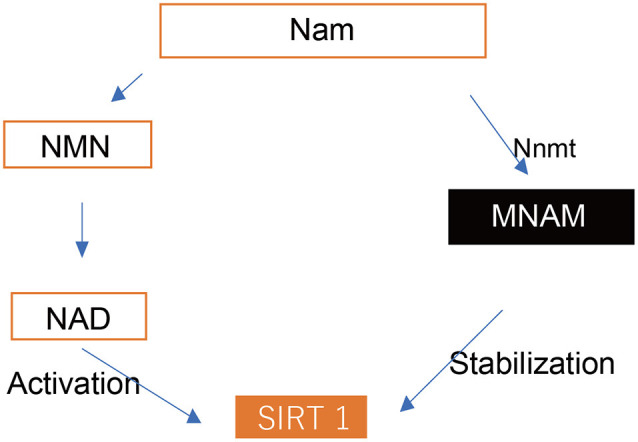
Cascade of Sirt1 activation. SIRT 1, Sirtuin 1; Nam, nicotinamide; NMN, nicotinamide mononucleotide; NAD, nicotinamide adenine dinucleotide; Nnmt, nicotinamide N-methyltransferase; MNAM, N^1^-methylnicotinamide.

Sirtuins (Sirt) are a family of nicotinamide adenine dinucleotide (NAD^+^)-dependent protein deacetylases that are known to extend the lifespan of lower organisms (Finkel et al., 2009). *Sirt1* expression plays critical roles in mammalian health and disease development and is often associated with the most complex physiological processes, including metabolism, the onset of cancer, and aging (Revollo and Li, 2013). At the cellular level, *Sirt1* controls deoxyribose nucleic acid (DNA) repair and apoptosis, circadian clocks, inflammatory pathways, insulin secretion, and mitochondrial biogenesis (Haigis and Sinclair, 2010; Chalkiadaki and Guarente, 2012). It also modulates apoptosis in response to oxidative and genotoxic stress in neurodegenerative diseases and ARHL (Someya et al., 2009; Donmez and Outeiro, 2013). Additionally, *Sirt1* may act as a sensor for metabolic adaptation to nutritional states since it is regulated by the availability of its substrate, NAD^+^. Thus, the enzymes involved in the NAD^+^ metabolic pathways may regulate metabolism through *Sirt1* (Chalkiadaki and Guarente, 2012). Nicotinamide N-methyltransferase (Nnmt) is an example of such an enzyme and regulates adipose tissue energy expenditure partly through induction of global changes in histone methylation and increased NAD^+^ content (Kraus et al., 2014). Nnmt methylates nicotinamide (NAM), a form of vitamin B3, to produce N^1^-methylnicotinamide (MNAM; schema, Figure 1). In humans, the expression of MNAM positively correlates with insulin resistance (Kannt et al., 2015). Additionally, MNAM possesses anti-inflammatory and antithrombotic properties and increases lifespan through the exertion of mitohormetic effects (Schmeisser et al., 2013). Both Nnmt and MNAM increase SIRT1 protein expression independently of its mRNA levels. SIRT1 is necessary for the exhibition of the metabolic effects of Nnmt and MNAM. Moreover, Nnmt and MNAM regulate the ubiquitin-proteasome degradation of SIRT1. Interestingly, expression of Nnmt is increased in B6 mice under the caloric restriction (CR) condition. This may promote SIRT3 expression, which is a mitochondrial Sirt that activates the glutathione-mediated mitochondrial antioxidant defense system. Thereby, several metabolic effects of CR may be mediated *via* Nnmt and SIRT3 expression enhancement (Someya et al., 2010). Thus, CR has implications in extending the lifespan and in the onset-delay of age-related diseases such as ARHL in mammals (Someya et al., 2010). Conversely, consumption of a high-fat diet (HFD) causes a decrease in SIRT1 expression (Wang et al., 2011; Chalkiadaki and Guarente, 2012) and damages metabolic changes such as glucose intolerance, insulin resistance, hepatic lipid accumulation and inflammation, and macrophage accumulation in the adipose tissue. This results in enhanced mitochondrial function, shortening of the lifespan (Chalkiadaki and Guarente, 2012), and early onset of ARHL in C57BL/6 (B6) and CBA/J mice (Le Prell et al., 2011; Du et al., 2012). Furthermore, elevated hearing thresholds during aging and a significant reduction in SIRT1 expression have been observed in the cochlea and auditory cortex of B6 mice (Keithley et al., 2004; Xiong et al., 2014). Hong et al. (2015) revealed the beneficial effects of dietary MNAM supplementation in B6 mice fed with an HFD as well as in humans. They reported that MNAM increased SIRT1 protein expression *in vivo* (Pfluger et al., 2008) and led to the exertion of systemic effects, such as significant lowering of serum cholesterol levels and changes in the lipoprotein profile (Hong et al., 2015). As it has been previously demonstrated that MNAM increases SIRT1 protein expression levels (Hong et al., 2015), these findings have led to the development of SIRT activators as a potential strategy to delay aging and age-related diseases in humans. However, use of a prevention therapy *via* MNAM supplementation for the delay of ARHL has not been reported. Therefore, we investigated its expression and roles in hearing function and the associated morphological changes in the cochlea using HFD, HFD + 1% MNAM (HFD-MNAM), and low-fat diet (LFD) aging B6 mice.

## Materials and Methods

### Animals

**Figure 2:**
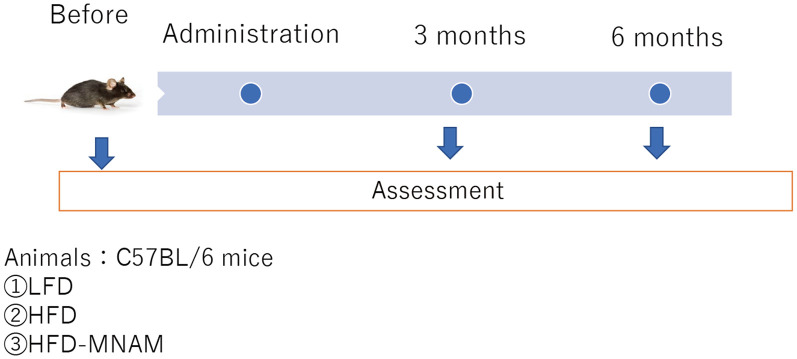
Schedule of experiments. LFD, low-fat diet; HFD, high-fat diet; HFD-MNAM, high-fat diet plus 1% N^1^-methylnicotinamide.

Thirty normal, 4-week-old, male B6 mice (*Mus musculus)* were purchased from Kyudo Company Limited (Kumamoto, Japan) and were randomly allocated into the following experimental groups: the HFD group, HFD-MNAM group, or LFD group (10 mice/group); subsequently, each group was further divided into the following subgroups (five mice/subgroup): 3-month-old and 6-month-old mice groups (Figure 2). Each subgroup was defined as YHFD, YHFD-MNAM, and YLFD for the 3-month-old mice groups and OHFD, OHFD-MNAM, and OLFD for the 6-month-old mice groups. The only criterion used was a matched group mean weight. The animals were housed in an air-conditioned room maintained at a temperature of approximately 25°C with an approximate humidity of 50%. After 1 week of acclimatization, the mice were fed with an irradiated LFD (Low-Fat Diet 32, CLEA Japan Inc., Tokyo, Japan), HFD (High-Fat Diet 32, CLEA Japan Inc.), or HFD supplemented with 1% MNAM (TCI America, Portland, OR, USA). HFD + 1% MNAM was prepared by grinding the feed in a blender and by mixing powdered MNAM at 1% wt./wt. The concentration of MNAM was determined by the previous study (Hong et al., 2015) and our preliminary study (data not shown). The mice were housed group-wise with each type of food and water available *ad libitum*. A fresh diet was prepared weekly. The LFD, HFD, and HFD + 1% MNAM amounts were adjusted appropriately based on the weight of mice. All animal experiments were approved by the Committee on the Use and Care of Animals (Kumamoto University, Japan) and were performed according to accepted veterinary standards (Number: H30-079).

### Body Weight and Blood Examination

Body weights were measured at baseline (pre-treatment) and 3 and 6 months after the commencement of the experiment. A venous blood sample was obtained from the tail and was measured using a complete clinical chemistry analyzer (BioMajesty^TM^ JCA-BM6050, JEOL Limited, Tokyo, Japan) 6 months after experiment initiation.

The mice were euthanized by cervical dislocation and were fixed *via* cardiac perfusion using 4% paraformaldehyde (PFA) in phosphate-buffered saline (PBS), and the inner ears were dissected from the adult temporal bones and decalcified in 0.5 M ethylenediaminetetraacetic acid (EDTA)/PBS. The tissues were embedded in the optimal cutting temperature medium (Sakura Finetek Japan, Tokyo, Japan) and were serially cryosectioned at a thickness of 12 μm for further examination.

### Immunohistochemistry

For the detection of antigens, the following primary antibodies and dilutions were used: anti-SIRT1 (1:100; cat. no. 13161-1-AP; Proteintech, Rosemont, IL, USA), anti-SIRT3 (1:200; cat. no. orb247889; Biorbyt, Cambridge, United Kingdom), anti-Na, K-ATPase 1 alpha1 (1:200; cat. no. NB300-146; Novus, Centennial, CO, USA), and anti-Connexin26 (1:200; cat. no. 710500; Life Technologies, Carlsbad, CA, USA). Fluorophore-conjugated secondary antibodies were used at a dilution of 1:500. Primary antibody labeling was performed at 4°C overnight after blocking in 10% goat or donkey serum for 10 min in PBS. Secondary antibody labeling was performed at 25°C for 1 h. Hoechst 33258 dye (Molecular Probes, Eugene, OR, USA) was used for 10 min to perform nuclear staining. The samples were examined under the BZ-9000 fluorescence microscope (Keyence, Osaka, Japan).

### Hair Cell Count

To study the surface morphology of the cochleae, the mice were fixed *via* cardiac perfusion with PFA/PBS under anesthesia, and the bony capsule and lateral wall of the cochlea were removed. Texas Red-X phalloidin (1:100; Molecular Probes) was applied for 30 min, and the surface morphology of the cochleae was examined under the BZ-9000 fluorescence microscope (Keyence). Five randomly selected surface images of the organ of Corti (OCs) in every turn of the cochlea were acquired at a 40× magnification in each group. The numbers of outer hair cells (OHC) and inner hair cells (IHC) in a 140-μm basal segment of the basilar membrane were calculated in each group. Only the hair cells with an intact stereocilia bundle and a cuticular plate were counted in every turn of the cochlea as per previously described methods (Miwa et al., 2020). A second researcher confirmed the accuracy of the measurements.

### Spiral Ganglion Cell Count

To assess the number of spiral ganglion cells (SGCs), the samples on the slides were labeled with the anti-beta III tubulin (Tuj1) antibody (1:200; MMS-435P, Covance, Princeton, NJ, USA), and the nuclei were counterstained using Hoechst 33258 dye (Molecular Probes). Three randomly selected images were acquired in every turn of the cochlea in each group. We classified the cells that were positive for both Tuj-1 and Hoechst staining in Rosenthal’s canal as SGCs. We counted the SGCs at the basal, in the middle, and at the apical turns in three randomly selected sections per cochlea and marked the counted cells on the images to avoid double counting using the ImageJ software (NIH, Framingham, MA, USA) as per previously described protocols (Yamada et al., 2015). The accuracy of the analysis was confirmed by an independent second researcher.

### Measurement of the Thickness of the Stria Vascularis

The average thickness of the stria vascularis was measured by analyzing the images of the sections containing the midmodiolar region using the ImageJ software (NIH) as per previously described methods (Miwa et al., 2019, 2020).

### ELISA

Each cochlear sample was homogenized using the Sonifier S-250A analog ultrasonic processor (Branson, Danbury, CT, USA). Protein concentrations were measured using a bicinchoninic acid protein assay kit (Thermo Fisher Scientific, Rockford, IL, USA) and were adjusted at the same concentration. Tissue lysates were assayed by using a mouse enzyme-linked immunosorbent assay (ELISA) SIRT1 kit (ab206983; Abcam, Cambridge, United Kingdom) for single protein quantification by following the manufacturer’s instructions. The plates were read on the Multiskan FC microplate reader (Thermo Fisher Scientific) at 450 nm.

### Western Blotting (WB) Analysis

To confirm the results of the ELISA technique, the same lysates were separated by performing 12.5% sodium dodecyl sulfate-polyacrylamide gel electrophoresis and were detected using the following primary antibodies: anti-SIRT1 (1:1,000; Proteintech), horseradish peroxidase-conjugated secondary antibodies (Bio-Rad, Hercules, CA, USA) against primary antibodies, and horseradish peroxidase-conjugated anti-β-actin (PM053-7; MBL, Nagoya, Japan) at a dilution of 1:5,000. The signals were visualized using an electrochemiluminescence system (Bio-Rad). The detected bands were analyzed using the ImageJ software (NIH); β-actin was used as the internal loading control.

### Real-Time Quantitative Reverse Transcription-Polymerase Chain Reaction (qRT-PCR)

Using a microRNA extraction kit (QIAGEN, Valencia, CA, USA), total RNA was extracted from each sample, quantified using the GeneQuant100 system (GE Healthcare, Amersham, UK), and diluted as required to achieve uniform concentrations. Complementary DNA (cDNA) was then synthesized using the One-Step PrimeScript RT-PCR Kit (Takara Bio, Otsu, Japan) according to the manufacturer’s instructions using primers for *Sirt1* and glyceraldehyde-3-phosphate dehydrogenase (*GAPDH*) (Applied Bionics, Foster City, CA, USA). The targets were amplified using the Takara Dice TP960 system by subjecting the samples to over 40 cycles of denaturation at 95°C for 15 s and by annealing them at 60°C for 1 min. Relative gene expression was calculated by generating a standard curve and was normalized to the *GAPDH* expression signal.

### Metabolome Analysis

Each mouse cochlear sample was dissected and washed using PBS and then preserved at −80°C until extraction. Metabolites were extracted using methanol containing hexamethylenetetramine internal standard solution 1 at 25°C. The metabolome was analyzed using gas chromatography coupled with mass spectrometry (Shimadzu Company, Tokyo, Japan). Peak detection was performed using the multivariate data analysis software (Travers MS, Reifycs Inc., Tokyo, Japan) in a three-step manner; first, mass values were detected within each spectrum. In the second step, a chromatogram was constructed for each of the mass values which spanned over a certain time range. Finally, deconvolution algorithms were applied to each chromatogram to recognize the actual chromatographic peaks. The average peak height for each metabolite was used for analysis, and the relative amounts of the metabolites were quantified. Heat map analysis, enrichment analysis, principal component analysis (PCA), and pathway analysis, including the TCA cycle, were performed using MetaboAnalyst^1^ (Pang et al., 2020).

### Auditory Thresholds

Before the commencement of the study, we examined the Preyer reflex and examined whether all mice possessed normal hearing abilities (Böhmer, 1988). We assessed the hearing thresholds in the 3-month-old and 6-month-old mice subgroups after the MNAM treatment by evaluating the auditory brainstem responses (ABR) (System 3; Tucker–Davis Technologies, Alachua, FL, USA) as per previously described protocols (Miwa et al., 2019). The animals were anesthetized as per the methods described above. Electrodes were placed beneath the pinna of the test ear and at the vertex below the surface of the skin. The ground electrode was placed under the contralateral ear. Auditory thresholds were measured at 4, 8, 12, 20, and 32 kHz by measuring the ABRs (15-ms duration, 1-ms rise/fall time, and tone burst). For each recording, an average of 512 sweeps was calculated. The stimulus levels near the threshold were varied in 10-dB steps, and the threshold was defined as the lowest level at which waves in the ABR could be clearly detected by visual inspection.

### Statistical Analysis

The data are presented as mean ± standard de*via*tion. A one-way analysis of variance (ANOVA) along with *ad hoc* Tukey–Kramer test (for all quantification experiments) was performed for the statistical analyses. *P* ≤ 0.05 was considered significant. All statistical analyses were performed using EZR (Saitama Medical Center, Jichi Medical University, Saitama, Japan), which is a graphical user interface of R (The R Foundation for Statistical Computing, Vienna, Austria). Precisely, it is a modified version of the R commander that has been designed to include statistical functions that are frequently used in biostatistics.

## Results

### HFD-Fed Mice Exhibit Marked Increase in Body Weight, Serum Cholesterol, Triglyceride, and Blood Glucose Levels

There were no differences in water and food consumption among the three groups. The bodyweight of the LFD-fed mice steadily increased throughout the 6-month observation period. The HFD-fed mice exhibited considerable weight gain (Supplementary Figures 1A,B) as expected. Although considerable differences were not observed among the YHFD, YHFD-MNAM, and YLFD groups, the body weights of the OHFD mice markedly increased compared with both the OLFD mice and OHFD-MNAM mice groups (Supplementary Figures 1A,B).

Differences in the results of blood analysis across all groups during the study period are presented in Supplementary Figure 1C. The alanine aminotransferase levels in the OHFD mice were markedly higher than those in the other two groups (Supplementary Figure 1C). More sustained effects were observed on the lipid and cholesterol levels in this group. The OHFD mice presented with significantly higher serum cholesterol and triglyceride levels compared with the other groups, probably because the LFD and MNAM supplementation included with the HFD prevented such increases in these groups (Supplementary Figure 1C). Blood glucose levels in these mice were markedly higher than those in the other two groups (Supplementary Figure 1C).

### HFD-Fed Mice Present With Severe Hearing Loss

**Figure 3:**
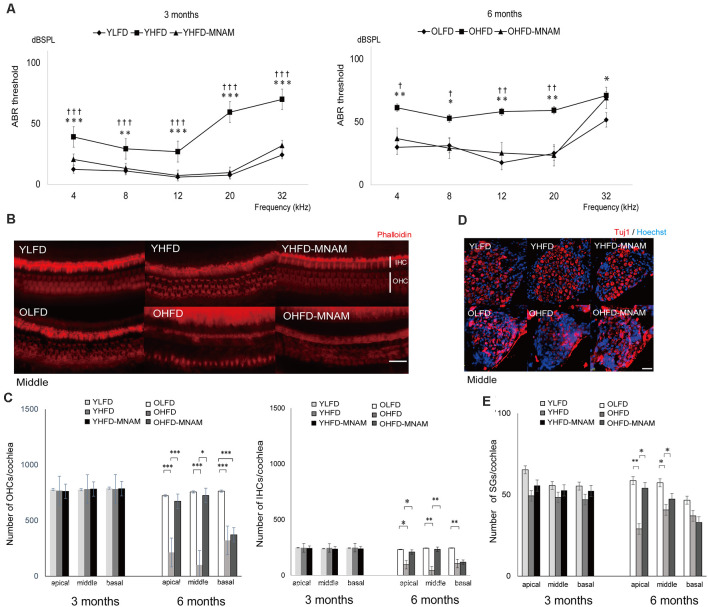
The onset of age-related hearing loss (ARHL) and effect of MNAM supplementation on ARHL: developmental changes in auditory thresholds, hair cell counts, and spiral ganglion cell counts in the three tested mice groups. **(A)** The ABR recording results at 3 and 6 months after commencement of the experiment. All groups developed age-related hearing loss at 6 months, rather than at 3 months (YLFD: *p* = at 4 kHz, *p* = at 8 kHz, *p* = at 12 kHz, *p* = at 20 kHz, and *p* = at 32 kHz, YHFD: *p* = at 4 kHz, *p* = at 8 kHz, *p* = at 12 kHz, *p* = at 20 kHz, and *p* = at 32 kHz, YHFD-MNAM: *p* = at 4 kHz, *p* = at 8 kHz, *p* = at 12 kHz, *p* = at 20 kHz, and *p* = at 32 kHz). The YHFD and OHFD developed severe hearing loss compared with the YLFD and OLFD mice (YHFD vs. YLFD: *p* < 0.001 at 4 kHz, *p* = 0.006 at 8 kHz, *p* < 0.001 at 12 kHz, *p* < 0.001 at 20 kHz, and *p* < 0.001 at 32 kHz, OHFD vs. OLFD: *p* = 0.003 at 4 kHz, *p* = 0.01 at 8 kHz, *p* = 0.003 at 12 kHz, *p* = 0.003 at 20 kHz, and *p* = 0.03 at 32 kHz). All groups, *n* = 5, *p < 0.05, **p < 0.01, ***p < 0.001. In the YHFD-MNAM and OHFD-MNAM groups, hearing loss was prevented markedly compared with the YHFD and YLFD groups (YHFD-MNAM vs. YLFD-MNAM: *p* < 0.001 at 4 kHz, *p* = 0.004 at 8 kHz, *p* < 0.001 at 12 kHz, *p* < 0.001 at 20 kHz, and *p* < 0.001 at 32 kHz, OHFD-MNAM vs. OLFD: *p* = 0.01 at 4 kHz, *p* = 0.04 at 8 kHz, *p* = 0.006 at 12 kHz, *p* = 0.005 at 20 kHz, and *p* = 0.40 at 32 kHz). All groups, *n* = 5, ^†^*p* < 0.05, ^††^*p* < 0.01, ^†††^*p* < 0.001. **(B)** Phalloidin-staining of the HCs in a surface preparation in the middle turn at 3 and 6 months after experiment initiation. The OHFD mice developed greater OHC and IHC loss than the other two groups. The OHFD-MNAM mice exhibited lower OHC and IHC loss compared with the OHFD mice. **(C)** The OHC and IHC counts in all turns at 3 and 6 months after commencement of the experiment. The YHFD mice did not develop OHC and IHC loss, compared to the other two groups (YLFD; OHC: *p* = 0.10 at apical, *p* = 0.09 in the middle, *p* = 0.13 at basal, IHC: *p* = 0.78 at apical, *p* = 0.61 in the middle, *p* = 0.43 at basal, YHFD-MNAM; OHC: *p* = 0.63 at apical, *p* = 0.10 in the middle, *p* = 0.62 at basal, IHC: *p* = 0.34 at apical, *p* = 0.23 in the middle, *p* = 0.41 at basal). The OHFD mice developed greater OHC and IHC loss than the other two groups (OLFD; OHC: *p* < 0.001 in all turns, IHC: *p* = 0.04 at apical, *p* = 0.008 in the middle, *p* = 0.002 at basal, OHFD-MNAM mice; OHC: *p* < 0.001 at apical, *p* = 0.01 in the middle, *p* = 0.67 at basal, IHC: *p* = 0.04 at apical, *p* = 0.009 in the middle, *p* = 0.29 at basal). All groups, *n* = 5, *p < 0.05, **p < 0.01, ***p < 0.001. **(D)** The immunostaining of the SGCs using the anti-Tuj1 antibody in the middle turn at 3 and 6 months after commencement of the experiment. The OHFD mice developed greater SGC loss than the other two groups. The OHFD-MNAM mice exhibited lower SGC loss compared with the OHFD mice. **(E)** The SGC counts in all turns at 3 and 6 months after commencement of the experiment. There were no significant differences in SGC numbers between YLFD and YHFD (*p* = 0.09 at apical, *p* = 0.28 in the middle, *p* = 0.10 at basal), and between YHFD and YHFD-MNAM mice (*p* = 0.31 at apical, *p* = 0.30 in the middle, *p* = 0.07 at basal). The OHFD mice developed greater SGC loss than the OLFD mice, except for the basal turn (*p* = 0.002 at apical, *p* = 0.03 in the middle, *p* = 0.07 at basal). The OHFD-MNAM mice exhibited less SGC loss than the OHFD mice (*p* = 0.01 at apical, *p* = 0.01 in the middle, *p* = 0.21 at basal). All groups, *n* = 5, *p < 0.05, **p < 0.01, ***p < 0.001 (Bars: 100 μm; YLFD, low-fat diet-fed 3-month-old mice; OLFD, low-fat diet-fed 6-month-old mice; YHFD, high-fat diet-fed 3-month-old mice; OHFD, high-fat diet-fed 6-month-old mice; YHFD-MNAM, high-fat diet plus 1% N^1^-methylnicotinamide-fed 3-month-old mice; OHFD-MNAM, high-fat diet plus 1% N^1^-methylnicotinamide-fed 6-month-old mice; OHCs, outer hair cells; IHCs, inner hair cells; SGCs, spiral ganglion cells; anti-Tuj1, anti-beta III tubulin).

ABR, an objective electrophysiological measure of hearing function, was used to monitor the progression of ARHL. The ABR recordings revealed that the YHFD and OHFD B6 mice developed severe hearing loss compared with the other two groups (Figure 3A and Supplementary Figures 2A–C). The average thresholds exhibited by the YHFD mice were markedly higher than those exhibited by the YLFD mice and the YHFD-MNAM mice at all examined frequencies (Figure 3A and Supplementary Figures 2A–C). The average thresholds exhibited by the OHFD mice were markedly higher than those exhibited by both the OLFD mice and OHFD-MNAM mice at all examined frequencies except for 32 kHz (Figure 3A and Supplementary Figures 2A–C).

### The Cochlea of HFD-Fed Mice Exhibit Alterations Indicating Hearing Loss and These Cochlear Alterations Are Absent in Mice Supplemented With MNAM

To validate the ABR hearing test results, we performed histological analysis on cochlear tissue sections. There was no apparent OHC loss, IHC loss, or SGC loss in any of the turns in the YHFD, YHFD-MNAM, and YLFD mice (Figures 3B–E). However, the OHFD mice exhibited a remarkable loss of OHCs and IHCs in all cochlear turns compared to the OLFD mice (Figures 3B,C). Moreover, in the OHFD-MNAM mice, the prevention of OHC and IHC loss was observed in all turns except for the basal turn (Figures 3B,C). The OHFD mice showed remarkable loss of SGCs in all cochlear turns except for the basal turn compared with the OLFD mice (Figures 3D,E). In the OHFD-MNAM mice, the prevention of SGC loss was observed in all turns (Figures 3D,E).

**Figure 4:**
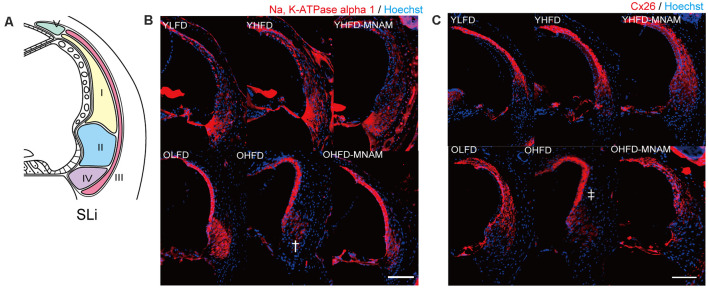
Morphological analysis of the spiral ligament (SLi): age-related SLi alterations are observed in OHFD mice. **(A)** The schema of the SLi. The fibrocytes of SLi are divided into five types. **(B)** The immunostaining of types II and IV in the SLi using an anti-Na, K-ATPase alpha 1 antibody at 6 months after experiment initiation. The dagger symbol indicates a decrease in Na, K-ATPase alpha 1 expression in the cochlea of OHFD mice after the commencement of the experiment. **(C)** The immunostaining of type I and V cells in the SLi using the anti-Cx26 antibody at 3 and 6 months after experiment initiation. The double dagger symbol indicates a decrease in Cx26 expression in the cochlea of the OHFD mice after the commencement of the experiment (Bars: 100 μm; YLFD, low-fat diet-fed 3-month-old mice; OLFD, low-fat diet-fed 6-month-old mice; YHFD, high-fat diet-fed 3-month-old mice; OHFD, high-fat diet-fed 6-month-old mice; YHFD-MNAM, high-fat diet plus 1% N^1^-methylnicotinamide-fed 3-month-old mice; OHFD-MNAM, high-fat diet plus 1% N^1^-methylnicotinamide-fed 6-month-old mice; OCs, the organ of Corti; SGC, spiral ganglion cells; SLi, spiral ligament; SV, stria vascularis).

The cochlea of YHFD mice showed slight degeneration of spiral ligament (SLi) cells (types II, IV, and V: Na, K-ATPase alpha 1 expression, type I: Cx26 expression) compared with the YLFD mice (Figures 4A–C). This degeneration of the SLi cells was not observed in the YHFD-MNAM mice (Figures 3A–C). Moreover, these SLi cells exhibited greater degeneration in the cochlea of OHFD mice compared with the OLFD mice (Figures 4A–C). Furthermore, this degeneration of the SLi cells was absent in the OHFD-MNAM mice (Figures 4A–C). The thickness of the stria vascularis was neither markedly different between YHFD, YLFD, and YHFD-MNAM mice nor was it different between OHFD, OHFD-MNAM, and OLFD mice (Supplementary Figure 3).

### MNAM-Treated HFD-Fed Mice Exhibit Higher Cochlear SIRT1 Protein Expression Compared With HFD-Fed Mice

**Figure 5:**
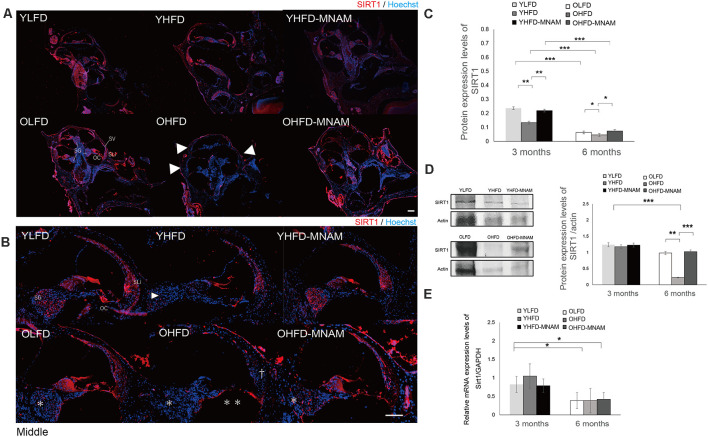
SIRT1 expression in the cochlea: the age-related SIRT1 downregulation of expression observed in OHFD mice is absent in OHFD-MNAM mice. **(A)** SIRT1 staining (red) of the whole cochlea at 3 and 6 months after commencement of the experiment. SIRT1 is prominently localized in the OCs, SGCs, and type II and IV SLi cells in all turns within the cochlea. The arrowheads indicate a decrease in SIRT1 expression in the cochlea of the OHFD mice. **(B)** SIRT1 staining (red) of the middle turn of the cochlea at 3 and 6 months after commencement of the experiment. The arrowhead indicates a decrease in SIRT1 expression in the SGCs in the cochlea of the YHFD mice after experiment initiation. The asterisks indicate a decrease in SIRT1 expression in the SGCs in the cochlea of the OHFD mice after experiment initiation. The double asterisk indicates a decrease in SIRT1 expression in the OCs in the cochlea of the OHFD mice after experiment initiation. The dagger symbol indicates a decrease in SIRT1 expression in the SLi in the cochlea of the OHFD mice after experiment initiation. **(C)** The SIRT1 protein expression in the cochlea quantitatively analyzed using ELISA at 3 and 6 months after commencement of the experiment. The SIRT1 protein expression analyzed *via* ELISA decreased with aging (*p* < 0.001 in each group). The YHFD and OHFD groups exhibited a decrease in the SIRT1 protein expression compared with the YLFD and OLFD groups (*p* = 0.002 and *p* = 0.04). In the YHFD-MNAM and OHFD-MNAM, decrease in the SIRT1 protein expression was lower than that in the YHFD and OHFD groups (*p* = 0.004 and *p* = 0.01; All groups, *n* = 5; *p < 0.05, **p < 0.01, ***p < 0.001). **(D)** The SIRT1 protein expression in the cochlea quantitatively analyzed using WB analysis at 3 and 6 months after commencement of the experiment. β-actin expression was used as a reference. The OHFD mice exhibited a greater decrease in the SIRT1 protein expression compared to the OLFD mice (*p* = 0.001). In the OHFD-MNAM group, decrease in the SIRT1 protein expression was less than that in the OHFD group (*p* < 0.001; All groups, *n* = 5; **p < 0.01, ***p < 0.001). **(E)** *Sirt1* mRNA expression in the cochlea quantitatively analyzed using qRT-PCR at 3 and 6 months after commencement of the experiment. *GAPDH* expression was used as a reference. *Sirt1* mRNA expression analyzed *via* ELISA decreased with aging (*p* = 0.03 in the YLFD and OLFD, *p* = 0.02 in the YHFD and OHFD, *p* = 0.04 in the YHFD-MNAM and OHFD-MNAM). The YHFD and OHFD mice did not exhibit a decrease in *Sirt1* mRNA expression compared with the YLFD and OLFD groups (*p* = 0.14 and *p* = 0.49). In the YHFD-MNAM and OHFD-MNAM groups, there were no decreases in *Sirt1* mRNA expression compared with those in the YHFD and OHFD groups (*p* = 0.11 and *p* = 0.45; Bars: 100 μm; *Sirt1*, Sirtuin 1; YLFD, low-fat diet-fed 3-month-old mice; OLFD, low-fat diet-fed 6-month-old mice; YHFD, high-fat diet-fed 3-month-old mice; OHFD, high-fat diet-fed 6-month-old mice; YHFD-MNAM, high-fat diet plus 1% N^1^-methylnicotinamide-fed 3-month-old mice; OHFD-MNAM, high-fat diet plus 1% N^1^-methylnicotinamide-fed 6-month-old mice; OCs, the organ of Corti; SGCs, spiral ganglion cells; SLi, spiral ligament; SV, stria vascularis; ELISA, enzyme-linked immunosorbent assay; GAPDH, glyceraldehyde-3-phosphate dehydrogenase; WB, western blotting; mRNA, messenger ribose nucleic acid; qRT-PCR, quantitative reverse transcription-polymerase chain reaction).

Using immunostaining assays, we determined whether cochlear SIRT1 levels increased with MNAM supplementation. Immunohistochemistry results revealed that the SIRT1 protein was present in the cochlea of YLFD mice, especially in types I, II, and V of SLi cells, OCs, including OHCs and IHCs, and SGCs (Figures 5A,B). In the OLFD mice, SIRT1 continued to be expressed and was well preserved in the OCs; however, it was decreased in the SLi cells and SGCs when compared with the YLFD mice (Figure 5B). The cochlea of the YHFD mice showed decreased SIRT1 expression in the OCs and SGCs Figures 5A,B), whereas, SIRT1 expression was not detected in the SLi cells, OCs, or SGC cochlea of the OHFD mice (Figure 5B), probably because the majority of the SLi cells, OHCs, IHCs, and SGCs was missing in these cochleae. Conversely, SIRT1 expression was preserved in the SLi cells, OCs, and SGCs of the YHFD-MNAM and OHFD-MNAM mice (Figure 5B). Moreover, immunostaining results revealed that SIRT3 expression followed the SIRT1 expression pattern in the inner ear (Supplementary Figure 4).

Further, to confirm these immunostaining results, we analyzed SIRT1 protein levels in inner ear tissues using WB analysis and ELISA. With aging, SIRT1 protein expression in the cochlea of OLFD mice was noticeably decreased (Figures 5C,D). SIRT1 protein expression in the OHFD mice was lower than that in the OLFD mice (Figures 5C,D). OHFD-MNAM mice demonstrated higher SIRT1 protein levels than the OHFD mice (Figures 5C,D), indicating that MNAM treatment prevented the decrease in SIRT1 protein levels in the cochlea of these mice. Although the mRNA expression of SIRT1 decreased with age in OHFD, OHFD-MNAM, and OLFD (Figure 5E), the decrease was not markedly different between the groups, even in OHFD, OHFD-MNAM, and OLFD mice (Figure 5E).

### Metabolome Analysis Reveals Differential Modulation of Various Metabolic Pathways in the Different Diet-Fed Mice Groups

**Figure 6:**
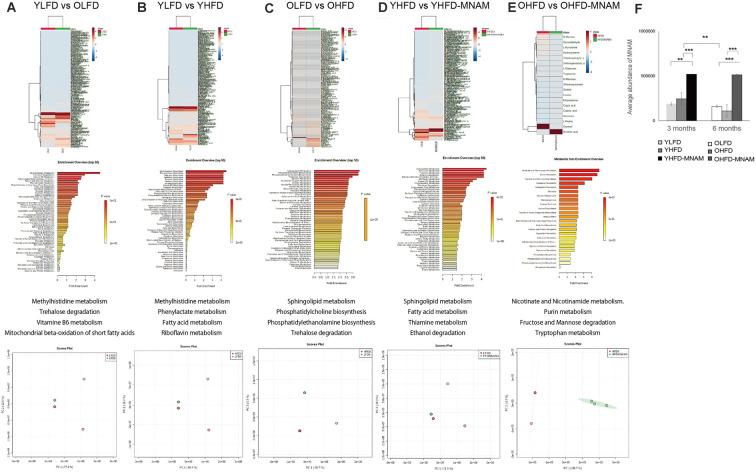
Metabolome heatmap, enrichment analysis, and principal component analysis (PCA). **(A)** Comparison between the cochlea of the YLFD and OLFD mice. The metabolic pathways related to methylhistidine metabolism, trehalose degradation, vitamin B6 metabolism, and the mitochondrial beta-oxidation of short-chain saturated fatty acids were activated in the cochlea of the OLFD mice. **(B)** Comparison between the cochlea of the YLFD and YHFD mice. The metabolic pathways related to glycolysis and aging were activated in the cochlea of the YHFD mice. **(C)** Comparison between the cochlea of the OLFD and OHFD mice. The metabolic pathways related to insulin resistance, obesity, non-alcoholic steatohepatitis, and aging showed greater activation in the cochlea of the OHFD mice. **(D)** Comparison between the cochlea of the YHFD and YHFD-MNAM mice. The metabolic pathways related to sphingolipid metabolism, fatty acid metabolism, thiamine metabolism, ethanol degradation, the mitochondrial beta-oxidation of short-chain saturated fatty acids, and riboflavin metabolism showed greater activation in the cochlea of the YHFD-MNAM mice. **(E)** Comparison between the cochlea of the OHFD and OHFD-MNAM mice. The metabolic pathways related to nicotine and nicotinamide metabolism, purine metabolism, fructose, and mannose degradation and tryptophan metabolism showed greater activation in the cochlea of the OHFD-MNAM mice. **(F)** MNAM metabolomic analysis peaks in the cochlea at 3 and 6 months after the commencement of the experiment. MNAM expression in both YHFD and OHFD decreased with aging (*p* = 0.002). MNAM expression in YHFD-MNAM increased compared to the expression in YLFD and YHFD (*p* = 0.001 and *p* < 0.001, respectively). MNAM expression in OHFD-MNAM increased compared to the expression in OLFD and OHFD (*p* < 0.001 and *p* < 0.001, respectively). YLFD, low-fat diet-fed 3-month-old mice; OLFD, low-fat diet-fed 6-month-old mice; YHFD, high-fat diet-fed 3-month-old mice; OHFD, high-fat diet-fed 6-month-old mice; YHFD-MNAM, high-fat diet plus 1% N^1^-methylnicotinamide-fed 3-month-old mice; OHFD-MNAM, a high-fat diet plus 1% N^1^-methylnicotinamide-fed 6-month-old mice. **p < 0.01, ***p < 0.001.

A metabolome enrichment analysis revealed that the metabolic pathways related to methylhistidine metabolism, trehalose degradation, vitamin B6 metabolism, and the mitochondrial beta-oxidation of short-chain saturated fatty acids were remarkably activated in the cochlea of OLFD compared to YLFD mice (Figure 6A; Supplementary Figure 5A). Furthermore, the metabolic pathways related to glycolysis and aging were markedly activated in the cochlea of YHFD compared to YLFD mice (Figure 6B; Supplementary Figure 5B). Conversely, insulin resistance, obesity, non-alcoholic steatohepatitis, and aging pathways were remarkably activated in the cochlea of OHFD compared to OLFD mice (Figure 6C; Supplementary Figure 5C). Further, we analyzed the differences in the metabolic pathways activated in the cochlea of YHFD and YHFD-MNAM mice. We found that the metabolic pathways related to sphingolipid metabolism, fatty acid metabolism, thiamine metabolism, ethanol degradation, mitochondrial beta-oxidation of short-chain saturated fatty acids, and riboflavin metabolism were selectively activated in the cochlea of YHFD-MNAM compared to YHFD mice (Figure 6D; Supplementary Figure 5D). Moreover, pathways related to nicotine and nicotinamide metabolism, purine metabolism, fructose, and mannose degradation, and tryptophan metabolism showed increased activation in the cochlea of OHFD-MNAM compared to that of OHFD mice (Figure 6E; Supplementary Figure 5E). Pathway analysis revealed that the TCA cycle was activated with a high-fat diet and aging, however, there were slight differences between OHFD and OHFD-MNAM (Supplementary Figures 5A–E). Additionally, MNAM expression in OHFD mice decreased compared to that in YHFD mice. MNAM expression in YHFD-MNAM and OHFD-MNAM exhibited a marked increase compared to other groups (Figure 6F).

## Discussion

### HFD-Fed Mice Exhibit ARHL and Decreased SIRT1 and SIRT3 Expression Levels

The increase in ABR thresholds associated with aging was markedly accelerated in the YHFD B6 mice compared with the YLFD mice. In OHFD B6 mice, there was a more remarkable loss of OHCs, IHCs, and SGCs in all cochlear turns as well as a loss of SLi cells (types I, II, V) as compared with the OLFD mice (Figures 3; Figure 4). These results suggest that HFD accelerates ARHL progression in B6 mice. These observations are consistent with results of previous studies, conducted using CBA/CaJ (Vasilyeva et al., 2009) and CD/1 mice (Hwang et al., 2013), which have reported that HFD induces oxidative stress, mitochondrial damage, and cellular apoptosis in the inner ear (Hwang et al., 2013). Conversely, Fujita et al. (2015) stated that ARHL was suppressed in HFD B6 mice. They speculated that the nutrients in the HFD (HFD32) were different from those present in a normal diet (CE-2); particularly, the level of the antioxidant and anti-inflammatory vitamin E (Jiang, 2014) is three times higher in HFD compared with a normal diet. They suggested that a combination therapy including antioxidants and vitamin E could reduce a noise-induced hearing loss (Le Prell et al., 2011) and prevent ARHL in B6 mice (Heman-Ackah et al., 2010). Here, LFD rather than a CE-2 was used as control. LFD and HFD are similar in the nutrient constitution, except for fat composition. Our results revealed that HFD induced a decrease in the expression of SIRT1 and SIRT3 proteins and mRNA levels in the cochlear samples. Results from immunohistology showed that their expression was decreased in the OHCs, IHCs, SGCs, and SLi cells in the cochlea of the OHFD mice (5 and Supplementary Figure 4). However, the SIRT1 and SIRT3 expression remained unaltered in the cochlea of the YHFD mice (Figure 5 and Supplementary Figure 4). These results were consistent with those of previous studies (Xiong et al., 2014), confirming that the inhibition of SIRT1 led to an increase in apoptosis in the mouse inner ear cell line (HEI-OC1) (Xiong et al., 2015). Furthermore, the metabolome enrichment analysis revealed that the cochlear metabolic pathways related to glycolysis and aging showed markedly higher activation in YHFD mice compared with YLFD mice (Figure 6B). However, insulin resistance, obesity, non-alcoholic steatohepatitis, and aging-related cochlear pathways showed increased activation in OHFD mice compared with those in OLFD mice (Figure 6C). SIRT1 regulates central metabolic functions, such as lipogenesis, protein synthesis, gluconeogenesis, and metabolic homeostasis, through deacetylation. Stress, particularly factors such as CR and HFD, alter Sirtuin activity that leads to significant alterations of certain intracellular processes; activation of reparation processes, increase in DNA stability, and elevation of metabolic rate and cell lifespan are observed. Our cochlear metabolome analysis results, including heatmap, enrichment analysis, PCA, pathway analysis (TCA cycle), were consistent with those reported in previous studies conducted on the investigation of liver metabolic profiles (Hong et al., 2015). Thus, our results suggested that during aging, HFD led to a SIRT decrease and caused metabolic changes in the inner ear, leading to early ARHL progression in B6 mice.

### MNAM Supplementation Increases SIRT1 and SIRT3 Expression Levels in the Cochlea and Suppresses ARHL in C57BL/6 Mice

We found that dietary MNAM supplementation exerted beneficial effects associated with the prevention of ARHL progression. MNAM protected the sensory organs and the lateral wall in the cochlea from damage in OHFD B6 mice. In OHFD-MNAM mice, the loss of OHCs, IHC, SGCs, and SLi cells (types I, II, IV, and V) was prevented in all cochlear turns except at the basal turn (Figures 3, 4), indicating protection conferred against HFD-related stress. MNAM exerted this protective effect by increasing the SIRT1 and SIRT3 protein expression levels in the cochlea. Immunostaining results revealed that SIRT1 and SIRT3 protein expression levels were detected in the OHCs, IHCs, SGCs, and SLi cells in YHFD-MNAM and OHFD-MNAM mice (Figure 5 and Supplementary Figure 4). However, this was not detected in the cochlea of OHFD mice. WB analysis and ELISA revealed significantly increased SIRT1 protein expression levels in the OHFD-MNAM mice compared with the levels in the OHFD mice (Figures 5C,D). Interestingly, no differences in the mRNA expression levels were observed among the three groups by qRT-PCR (Figure 5E). These results indicate that MNAM increases the SIRT1 protein expression levels independently of its mRNA levels. This observation is consistent with those reported in previous studies (Hong et al., 2015). Further, our findings suggest that dietary MNAM supplementation may activate SIRT3 expression in the cochlea. Previous studies have demonstrated that the activation of SIRT3 *via* the NAD^+^ precursor, nicotinamide riboside, conferred protection against noise-induced hearing loss (Brown et al., 2014). However, further analysis will be necessary to confirm this result, as we have not extensively examined the SIRT3 protein and mRNA expression levels in the cochlea.

Additionally, metabolome enrichment analyses revealed that the cochlear metabolic pathways related to insulin resistance, aging, non-alcoholic steatohepatitis, oxidative damage, and mitochondrial beta-oxidation were activated to a greater extent in YHFD mice than those in YHFD-MNAM mice. However, the cochlear metabolic pathways related to nicotine, mitochondrial beta-oxidation, glycolysis, aging, and oxidative damage were activated to a greater extent in the cochlea of OHFD mice as compared with those in OHFD-MNAM mice. Previous studies have suggested that MNAM regulates glucose, lipid, and cholesterol metabolism by stabilizing SIRT1 expression in the liver (Hong et al., 2015). Thus, our results suggest that MNAM supplementation provides beneficial effects on metabolic pathways in cochlear aging related to hearing function. Interestingly, the TCA cycle did not activate in OHFD-MNAM. Further studies are necessary.

### Adequate SIRT1 Expression May Play a Key Role in ARHL

In summary, our results revealed that HFD consumption-mediated downregulated expression of SIRT1 and SIRT3 and aging together lead to hearing loss. Additionally, MNAM-mediated cochlear upregulation of SIRT1 and SIRT3 protein expression levels exerted a preventive effect against the HFD- and aging-induced hearing loss. A similar result was obtained in a recent study conducted by the International Mouse Phenotyping Consortium. Their study demonstrated that young mice with homozygous *Sirt1* knockout mutation exhibited significantly increased ABR thresholds compared with B6 control mice^2^. However, conversely, Han et al. (2016) have reported that in hetero *Sirt1* transgenic B6 mice, the SIRT1 half deficiency reduces the age-related oxidative damage of the OHCs and SGCs while delaying the early onset of ARHL. This effect is caused by the enhancement of cochlear Foxo3a-mediated oxidative stress resistance. They hypothesized that since homozygous *Sirt1* mutant mice infrequently survived postnatally and were small in size presenting with developmental defects, it might be possible that the MRC Harwell study reflected higher ABR thresholds due to developmental defects present in the inner ear and/or central nervous system (Cheng et al., 2003). However, Alcendor et al. (2007) reported that while *Sirt1* overexpression increased apoptotic cell death in the heart and decreased cardiac function in mice, *Sirt1* inhibition protected rat cortical neurons against oxidative stress (Li et al., 2008). The same study reported that the moderate heart-specific overexpression of *Sirt1* protected the heart from oxidative stress induced by paraquat and increased catalase expression in mice (Alcendor et al., 2007). Thus, current reports on the roles of Sirtuins in extending health-span and lifespan are controversial.

Therefore, we speculated that “minimum” expression levels of SIRT1 were crucial for the prevention of ARHL progression. In agreement with this hypothesis, our results revealed that the decrease in SIRT1 expression levels induced *via* HFD consumption and aging in B6 aged mice resulted in ARHL. Furthermore, moderate SIRT1 expression levels induced *via* MNAM supplementation in HFD-fed and aged B6 mice did not lead to ARHL. However, a previous report has suggested that the half expression of SIRT1 did not lead to ARHL (Xiong et al., 2014), whereas the lack of SIRT1 expression led to hearing loss. Considering these findings together, we suggest that SIRT1 may act as a pro-aging molecule in the cochlear cells of B6 mice. However, there are no reports on the mechanism by which a moderate inner-ear-specific overexpression of SIRT1 influences the progression of ARHL. Further research is warranted to elucidate the relationship between the level of SIRT1 expression in the cochlea and the development of ARHL.

A limitation of our present study is that the progression of hearing loss was enhanced by HFD consumption. With an LFD consumption, there were no effects of MNAM on hearing loss detected in a preliminary study. Therefore, an HFD was selected for this study. HFD-fed mice might not be an appropriate model for studying ARHL. The overexpression of SIRT1 in the cochlea induced *via* MNAM supplementation or a genetic modification should be demonstrated in LFD-fed and aging B6 mice. Further research using an LFD ARHL mouse model is necessary. Additionally, our results may also be interpreted to suggest that MNAM solely acts as a preventive agent against ARHL. The etiologies of ARHL are due to multiple factors. Our study may have other signaling involved. Furthermore, our study showed that MNAM supplementation failed to prevent changes in the cochlear basal turn leading to ARHL. Differential MNAM concentration distribution in the cochlear turns may explain the lack of ARHL prevention in the basal turn. Studies measuring MNAM concentration in each cochlear turn may provide more evidence.

## Data Availability Statement

The raw data supporting the conclusions of this article will be made available by the authors, without undue reservation.

## Ethics Statement

The animal study was reviewed and approved by Committee on the Use and Care of Animals (Kumamoto University, Japan).

## Author Contributions

TM designed the research studies, conducted the experiments, acquired the data, analyzed the data, and wrote the manuscript.

## Conflict of Interest

The author declares that the research was conducted in the absence of any commercial or financial relationships that could be construed as a potential conflict of interest.

## Footnotes

^1^http://www.metaboanalyst.ca/

^2^http://www.mousephenotype.org/phenoview/?gid%2520=%2520363&qeid%2520=%2520MP:0004738&ctrl%2520=%25202357729&pt%2520=%25200.0001

## Abbreviations

ARHL: age-related hearing loss
NAD+: nicotinamide adenine dinucleotide
MNAM: N^1^-methylnicotinamide
HFD: high-fat diet
LFD: low-fat diet
SIRT: Sirtuins
Nnmt: nicotinamide N-methyltransferase
NaM: nicotinamide
CR: caloric restriction
PFA: paraformaldehyde
EDTA: ethylenediaminetetraacetic acid
PBS: phosphate-buffered saline
Tuj1: anti-beta III tubulin
SGCs: spiral ganglion cells
GAPDH: glyceraldehyde-3-phosphate dehydrogenase
cDNA: complementary DNA
ANOVA: analysis of variance
ABR: auditory brainstem responses
SLi: spiral ligament cells
OC: the organ of Corti
WB: western blotting
ELISA: enzyme-linked immunosorbent assay.
