# Supplementary material for: Protective Effects of N1-Methylnicotinamide Against High-Fat Diet- and Age-Induced Hearing Loss via Moderate Overexpression of Sirtuin 1 Protein
Source: Front Cell Neurosci. 2021 Apr 6;15:634868. doi: 10.3389/fncel.2021.634868 (PMC8055820; doi:10.3389/fncel.2021.634868)
Supplement: Supplementary file 1 [file Image_1.pdf]

## *Supplementary Material*

# 1 Supplementary Figures and Tables

## 1.1 Supplementary Figures

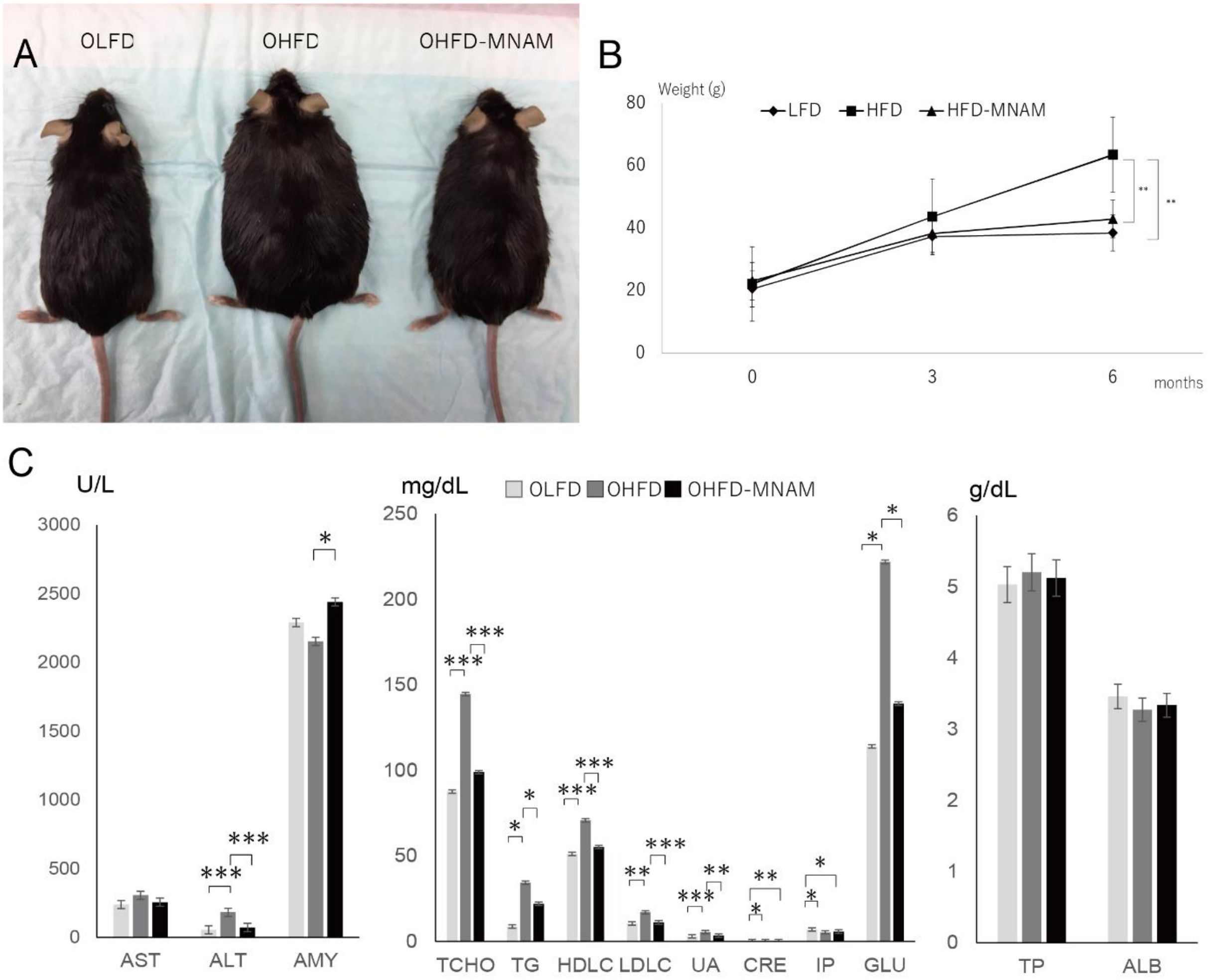


**Supplementary Figure 1. Differences in weight and blood analysis results**

(A) Images of the animals in each group at 6 months after experiment initiation.

(B) Body weights of the mice at 3 and 6 months after experiment initiation. The body weights of the YHFD mice did not significantly increase compared with those of the YLFD ($p = 0.10$) or the YHFD-MNAM mice ($p = 0.34$). The body weights of the OHFD mice significantly increased

compared with those of the OLFD ($p = 0.001$) and the OHFD-MNAM mice ($p = 0.003$; All groups, $n = 5$);

(C) Differences in the blood analysis results of all groups at 6 months after experiment initiation. The alanine aminotransferase (ALT), total cholesterol (TCHO), triglyceride (TG), high-density lipoprotein cholesterol (HDLC), low-density lipoprotein cholesterol (LDLC), uric acid (UA), and glucose (GLU) levels in the OHFD mice were significantly higher than those in the other two groups (vs OLFD; $p <0.001$, $p = 0.001$, $p = 0.02$, $p = 0.003$, $p = 0.01$, $p < 0.001$ and $p = 0.01$, vs OHFD-MNAM; $p < 0.001$, $p < 0.001$, $p = 0.02$, $p = 0.009$, $p = 0.007$, $p = 0.001$ and $p < 0.001$). The creatinine (CRE) and inorganic phosphorus (IP) levels in the OLFD mice were significantly higher than those in the other two groups (vs OHFD; $p = 0.03$ and $p = 0.003$, vs OHFD-MNAM; $p = 0.008$ and $p = 0.01$; All groups, $n = 5$; *: $p <0.05$, **: $p < 0.01$, ***: $p < 0.001$)

[LFD, low-fat diet; HFD, high-fat diet; HFD-MNAM, high-fat diet plus 1% $N^1$-methylnicotinamide; OLFD, low-fat diet-fed 6-month-old mice; OHFD, high-fat diet-fed 6-month-old mice; OHFD-MNAM, high-fat diet plus 1% $N^1$-methylnicotinamide-fed 6-month-old mice]

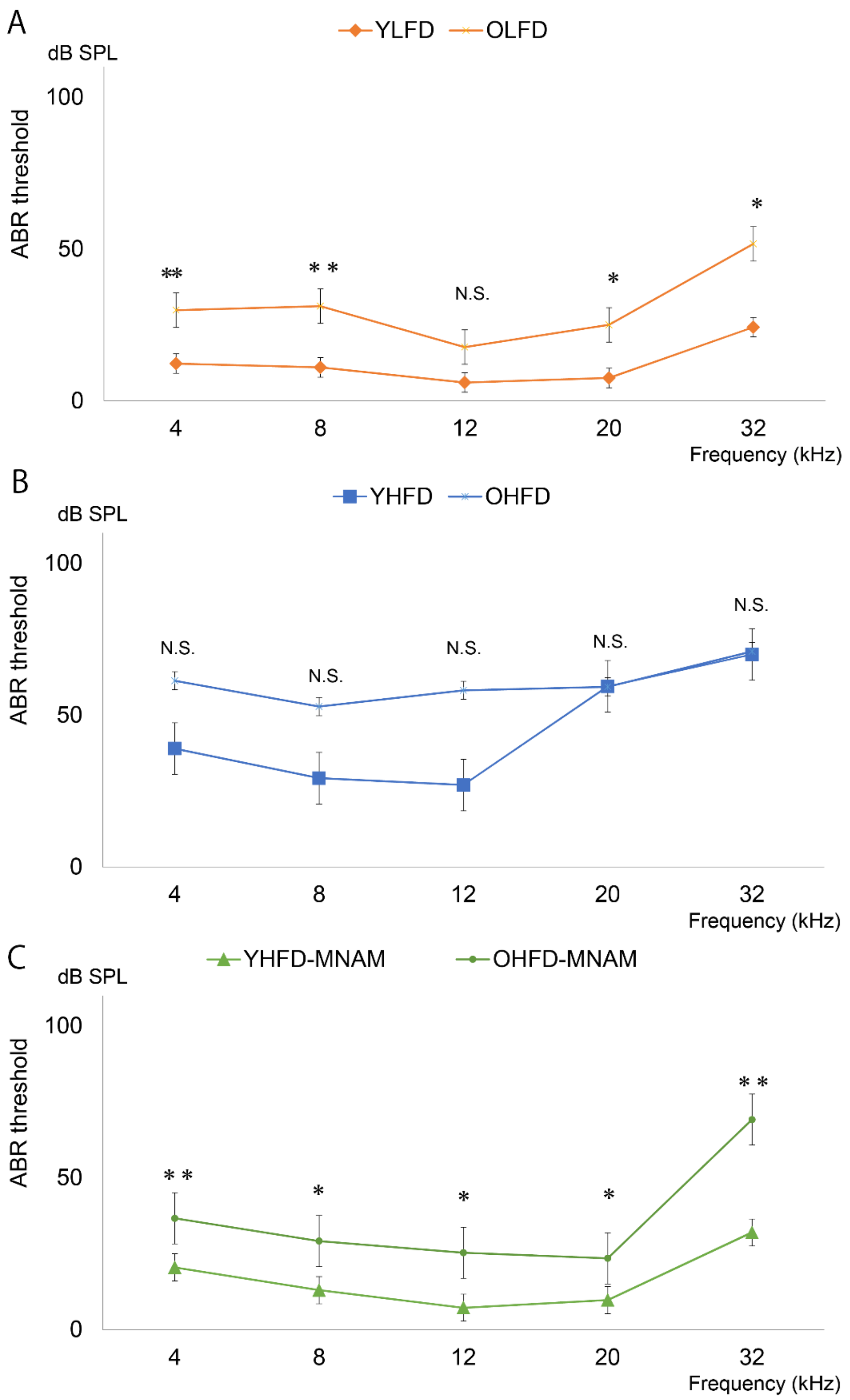


**Supplementary Figure 2. Auditory brainstem responses (ABR)**

[YLFD, low-fat diet-fed 3-month-old mice; OLFD, low-fat diet-fed 6-month-old mice; YHFD, high-fat diet-fed 3-month-old mice; OHFD, high-fat diet-fed 6-month-old mice; YHFD-MNAM, high-fat diet plus 1% $N^1$-methylnicotinamide-fed 3-month-old mice; OHFD-MNAM, high-fat diet plus 1% $N^1$-methylnicotinamide-fed 6-month-old mice]

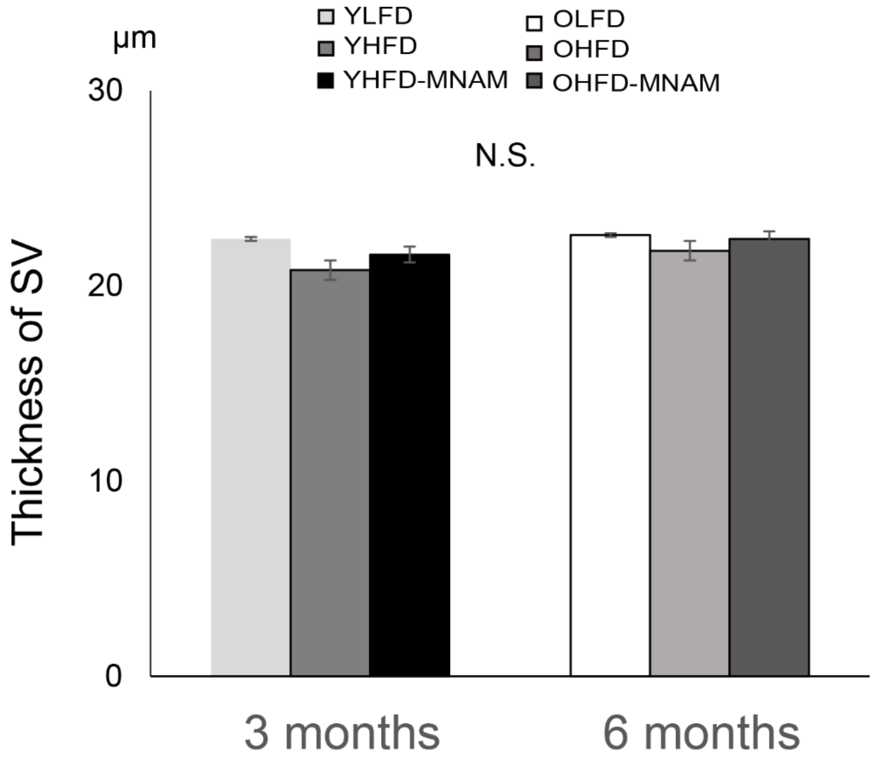


**Supplementary Figure 3. Thickness of Stria vascularis (SV)**

Analysis after immunostaining of SV revealed that there were no significant differences in thickness of SV among all groups.

[SV, stria vascularis; YLFD, low-fat diet-fed 3-month-old mice; OLFD, low-fat diet-fed 6-month-old mice; YHFD, high-fat diet-fed 3-month-old mice; OHFD, high-fat diet-fed 6-month-old mice; YHFD-MNAM, high-fat diet plus 1% $N^1$-methylnicotinamide-fed 3-month-old mice; OHFD-MNAM, high-fat diet plus 1% $N^1$-methylnicotinamide-fed 6-month-old mice]

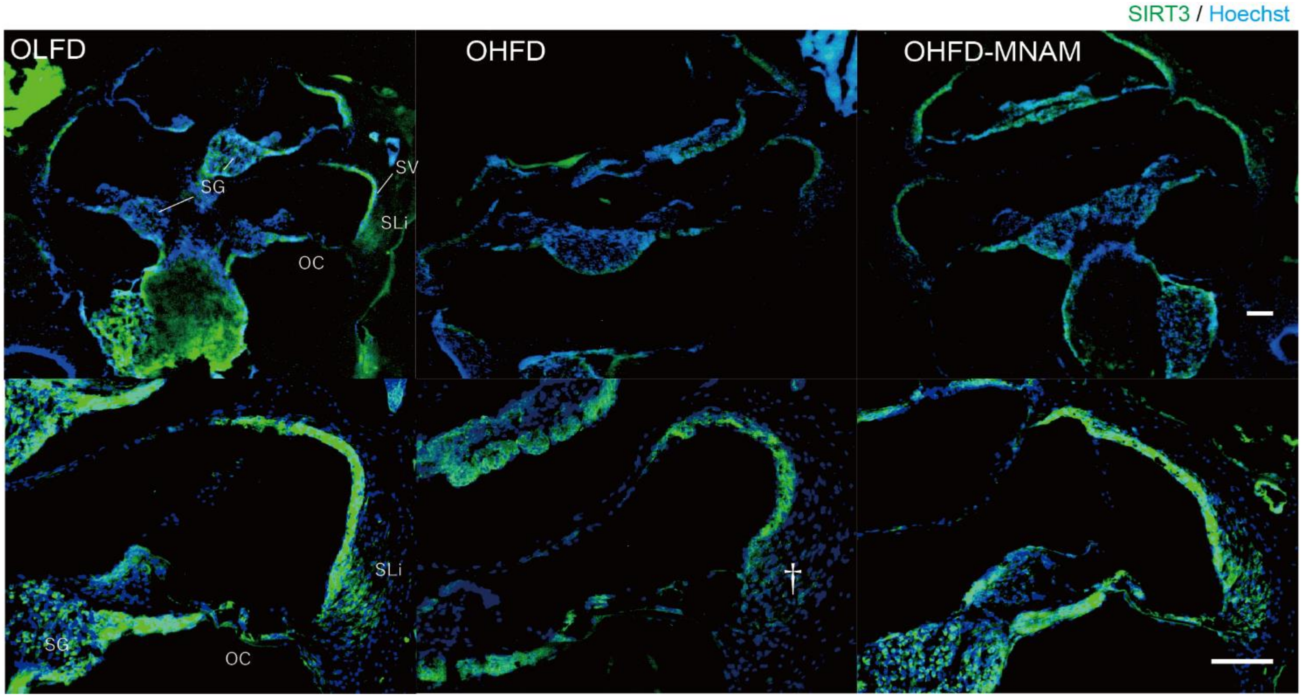


**Supplementary Figure 4. Sirt 3 expression in the cochlea**

SIRT3 staining (green) of the middle turn of the cochlea at 6 months after experiment initiation. The dagger symbol indicates a decrease in the SIRT3 protein expression in the SLi cells at 6 months after commencement of the experiment.

[SIRT3, Sirtuin 3; OCs, the organ of Corti; SGC, spiral ganglion cells; SLi, spiral ligament; SV, stria vascularis; OLFD, low-fat diet-fed 6-month-old mice; OHFD, high-fat diet-fed 6-month-old mice; OHFD-MNAM, high-fat diet plus 1% $N^1$-methylnicotinamide-fed 6-month-old mice]

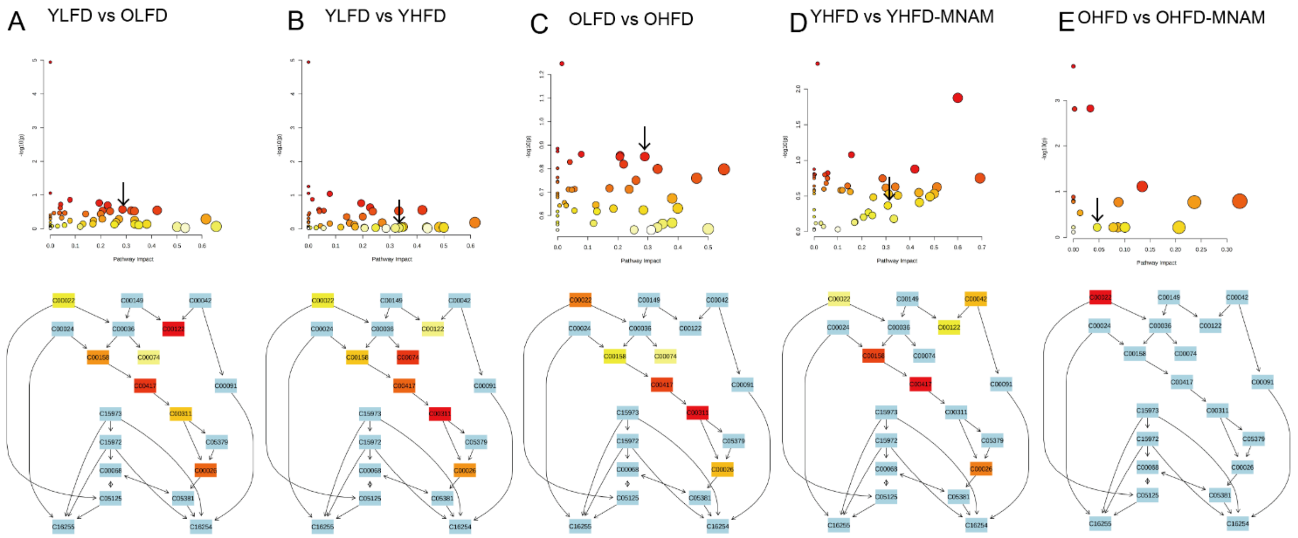


**Supplementary Figure 5. Metabolome pathway analysis and TCA cycle.**

(A-E) Comparison between the cochlea of the YLFD and OLFD mice (A), the YLFD and YHFD mice (B), the OLFD and OHFD mice (C), the YHFD and YHFD-MNAM mice (D) and the OHFD and OHFD-MNAM mice (E). Arrow indicated TCA cycle.

C00022: Pyruvic acid, C00024: Acetyl-CoA, C00158: Citrate, C00149: Malate, C00036: Oxaloacetate, C00074: Phosphoenolpyruvate, C00417: Aconitate, C00042: Succinate, C00122: Fumarate, C00091: Succinyl-CoA, C00311: Isocitrate, C05379: Oxalosuccinate, C00026: 2-Oxoglutarate

[YLFD, low-fat diet-fed 3-month-old mice; OLFD, low-fat diet-fed 6-month-old mice; YHFD, high-fat diet-fed 3-month-old mice; OHFD, high-fat diet-fed 6-month-old mice; YHFD-MNAM, high-fat diet plus 1% $N^1$-methylnicotinamide-fed 3-month-old mice; OHFD-MNAM, high-fat diet plus 1% $N^1$-methylnicotinamide-fed 6-month-old mice]
